# No improvement in socioeconomic inequalities in birthweight and preterm birth over four decades: a population-based cohort study

**DOI:** 10.1186/1471-2458-13-345

**Published:** 2013-04-15

**Authors:** Svetlana V Glinianaia, Rakesh Ghosh, Judith Rankin, Mark S Pearce, Louise Parker, Tanja Pless-Mulloli

**Affiliations:** 1 Institute of Health & Society, Newcastle University, Newcastle upon Tyne, England, UK; 2 Department of Public Health Sciences, University of California, Davis, USA; 3 Departments of Medicine and Pediatrics, Dalhousie University, Halifax, NS, Canada; 4 Institute of Health & Society, Newcastle University, Baddiley-Clark Building, Richardson Road, Newcastle upon Tyne, NE2 4AX, UK

**Keywords:** Low birthweight, Preterm birth, Socioeconomic status, Townsend deprivation score, Temporal trends

## Abstract

**Background:**

Birthweight and gestational age are associated with socioeconomic deprivation, but the evidence in relation to temporal changes in these associations is sparse. We investigated changes in the associations between socioeconomic status (SES) and birthweight and gestational age in Newcastle upon Tyne, North of England, during 1961–2000.

**Methods:**

We used population-based data from hospital neonatal records on all singleton births to mothers resident in Newcastle (births with complete covariate information n = 113,182). We used linear regression to analyse the associations between neighbourhood SES and birthweight over the entire 40-year period and by decade, and logistic regression for associations with low birthweight (LBW) and preterm birth, adjusting for potential confounders.

**Results:**

There was a significant interaction between SES and decade of birth for birthweight (p = 0.028) and preterm birth (p < 0.001). Socioeconomic gradients were similar in each decade for birthweight outcomes, but for preterm birth, socioeconomic disparities were more evident in the later decades [for 1961–70, odds ratio (OR) was 1.1, 95% CI 0.9, 1.3, for the most deprived versus the least deprived quartile, while for 1991–2000, the corresponding OR was 1.5, 95% CI 1.3, 1.7]. In each decade, there was a significant decrease in birthweight adjusted for gestational age for the most deprived compared to the least deprived SES group [1961–1970: –113.4 g (95% CI–133.0, –93.8); 1991–2000: –97.5 g (95% CI–113.0, –82.0)], while there was a significant increase in birthweight in each SES group over time.

**Conclusions:**

Socioeconomic inequalities did not narrow over the four decades for birthweight and widened for preterm birth. Mean birthweight adjusted for gestational age increased in all socioeconomic groups, suggesting an overall increase in fetal growth.

## Background

Birthweight and gestational age are major determinants of perinatal and infant mortality [1,2], with low birthweight (LBW) and preterm birth being associated with much higher risks of infant mortality [3], childhood morbidity and developmental disability [4,5]. Impaired fetal growth is also considered to contribute to adult diseases, such as diabetes and cardiovascular disease [6]. In developed countries, the overall rates of preterm birth and LBW increased over the last two decades of the 20th century [7-11] as well as later [12,13] as a result of the rise in multiple birth rates, but also due to an increase in preterm birth rates in singletons consequent to obstetric interventions at earlier gestation. In addition to the societal and family burden of increased mortality and disability linked with reduced birthweight and duration of pregnancy, there is a substantial cost to health services in providing appropriate care for these infants [14], which makes it an important public health issue.

The well-established associations of adverse perinatal outcomes, such as LBW and preterm birth, with socioeconomic deprivation [2,15-19] are mediated through maternal nutritional status, smoking and drinking habits, and exposure to environmental hazards etc [18,20-22]. Given the widening differences in overall mortality between the affluent and deprived groups in the United Kingdom [23], and persistent inequalities in perinatal mortality [24], it is important to monitor whether socioeconomic inequalities in birthweight and gestational age reduced over time as a result of wider access to health care and dramatic advances in prenatal care, improvement in people’s living conditions, nutrition and public health attempts to tackle socioeconomic health inequalities.

This population-based study aims to investigate decadal changes in birthweight and gestational age in relation to area-based socioeconomic status (SES) in Newcastle upon Tyne, UK, from 1961 to 2000. Compared to studies from larger areas over shorter periods [25-27], this study from a relatively small conurbation, with a rich industrial history and a stable population, covers a four-decade period. This allowed us to examine temporal changes in the associations between area deprivation and both birthweight and gestational age, which is not possible in studies using vital statistics birth record data.

## Methods

This population-based study included all singleton births to mothers resident in Newcastle upon Tyne, North of England, from 1961 to 2000 (population reduced from 336,000 in 1961 to 266,200 in 2001: http://www.newcastle.gov.uk/your-council/statistics-and-census-information/population-past-estimates-and-trends). Newcastle has transformed from a city with heavy industry and coal production in the early 1960s to a city with a predominantly service-based economy by 2000. The population structure of the region is characterised by a low percentage of ethnic minorities (2%) [28], and a relatively stable population with low levels of both inward and outward migration, including low residential mobility in pregnancy [29]. A major part of the database (over 1961–1992) was compiled for the historical Particulate Matter and Perinatal Events Research (PAMPER) study [30], with data for later years (1993–2000) being added. A description of the population and the construction of the PAMPER dataset, its completeness and accuracy has been reported previously [31]. Briefly, for 1961–92 we collected information on births from hospital neonatal records of the two major maternity hospitals at that time, which merged into one maternity hospital in 1993. We also obtained information on home births, which constituted approximately one-third of all births during the early 1960s (reducing to 0.4% in 1973) [31], from birth ledgers. Birth ledgers did not record information on birthweight, gestational age and other covariates, but helped in ascertaining the total number of births for the early 1960s. We also used several other local (Tyne and Wear Archives), regional (Regional Maternity Survey Office, Newcastle) and National (Office for National Statistics, UK) sources to construct and validate the database.

We defined LBW as a birthweight less than 2500 g; gestational age as time between the woman’s last normal menstrual period (LMP) and date of delivery; preterm birth as birth at less than 37 completed weeks of gestation. For consistency across the study period, and meaningful comparison of preterm birth and low birthweight rates between the decades, we excluded stillbirths at <28 completed weeks of gestation, even for births from October 1992 when the legal cut-off in gestational age for stillbirth was changed to 24 completed weeks in England and Wales. Births with a birthweight <500 g were also excluded if gestational age was unknown.

The Townsend Deprivation Score (TDS), an area-based measure of material deprivation incorporating the proportion of home ownership, car ownership, unemployment and overcrowding, was calculated at the enumeration district (ED) level using data from the 1971 (for 1961–76), 1981 (for 1977–1986) and 1991 (for 1987–1996) UK Census Surveys. ED is the finest geographical scale of census area (approximately 450 people in 200 households) in England and Wales for which census data were available. We identified EDs from the maternal residential postcode at birth (typically one postcode covers about 15 addresses) or grid reference (a unique spatial identifier commonly used in the UK that specifies a position on a map). We used the 2001 Census data for births between 1997 and 2000 to calculate TDS which were available at the Census ward level (census output areas covering about 120 households were allocated to postcodes, from which Census wards were obtained and the appropriate TDS were assigned) rather than ED level. We used TDS quartiles for this analysis, Q1 representing those resident in wards assumed to be the least deprived to Q4 representing those assumed to be most deprived. Since there can be considerable change in SES from one decade to the other [32], we calculated TDS quartiles separately for each decade to increase comparability.

There were 131,044 singleton births within the study area between 1961 and 2000; birthweight was available for 118,450 (90.4%) and gestational age for 116,185 (88.7%) births. Data on birthweight and gestational age were missing mainly for home births in the earlier period of the study as it was not recorded in birth ledgers. Information was also available on maternal residential address including postcode, maternal age, parity, TDS and infant sex. Infant sex was available for 131,033 births (>99.9%), maternal age, parity and TDS were available for 118,027 (90.1%), 118,371 (90.3%) and 128,459 (98.0%) births respectively. Multivariable models used 113,182 births for birthweight and 113,431 for gestational age outcomes.

We calculated mean birthweight and the proportions of LBW and preterm birth for each TDS quartile and plotted the trends. We used five-year moving averages to smooth the trends as there was considerable annual variation in each of the outcomes. In multivariable analyses, we fitted linear regression models to birthweight and logistic regression models to the dichotomous outcomes, LBW and preterm birth. We report regression coefficients for linear regression and odds ratios (ORs) for logistic regression with corresponding 95% confidence intervals (95% CI). Maternal age, parity and infant sex were included as categorical variables in the model with maternal age as five year groups (<20, 20–24, 25–29, 30–34, 35–39 and ≥40 years) and parity as primipara, one, two, three and ≥ four. TDS quartiles (with 25^th^, 50^th^ and 75^th^ percentile cut-offs) and decades of birth (1–1961–1970, 2–1971–1980, 3–1981–1990 and 4–1991–2000) were also fitted as categorical variables in the models. We chose to use decade of birth rather than individual years to avoid noise due to annual fluctuations in outcomes and to link in with the use of census data. A product term was used to examine whether there was an interaction between TDS quartiles and decade of birth in the adjusted model for each outcome. We also carried out separate analyses by decade. STATA, version 10.1 (StataCorp, College Station, TX, USA) was used for statistical analyses.

The PAMPER study received a favourable ethical opinion from the Sunderland Local Research Ethics Committee (SLREC 1071) to use the hospital neonatal records for 1961–1992. We also received ethical and Caldicott approval to use the birth records for 1993–2002 to extend the PAMPER birth record database. For this sub-analysis we used anonymised data only.

## Results

For the entire study period, there was a clear socioeconomic gradient with a higher risk of reduced birthweight (–95g, 95% confidence interval (CI)–103, –87), LBW (OR 1.6, 95% CI 1.4, 1.7) and preterm birth (OR 1.5, 95% CI 1.4, 1.6) for the most deprived compared to the least deprived TDS quartile.

**Figure 1:**
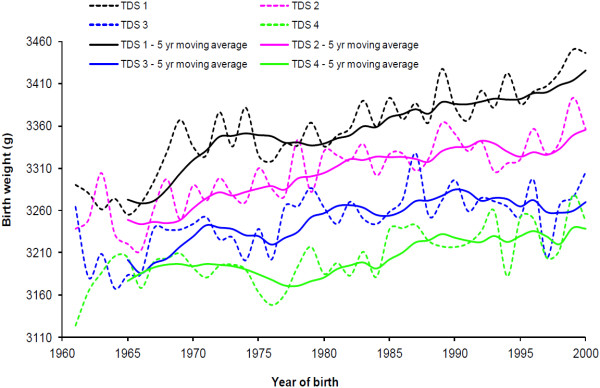
**Mean birthweight by area deprivation measured by Townsend deprivation score (TDS), quartiles, Newcastle upon Tyne 1961-2000.** The dotted lines are the observed (yearly) means for each TDS quartile, the solid lines are the five-year moving averages.

Overall, mean birthweight increased from 3227 g in 1961 to 3333 g in 2000 (Figure 1). Mean birthweight increased in all TDS groups, but the increase was 25% greater in the least deprived group from 3291 g in 1961 to 3447 g in 2000 compared to the most deprived group, from 3124 g in 1961 to 3247 g in 2000. Changes in mean birthweight for Q2 and Q3 were in between the two extreme groups. The mean birthweights (SD) for each TDS quartile by decade are shown in Table 1.

**Table 1:** Birthweight (mean, SD) and the percentage of low birthweight and preterm birth by decade and by area deprivation (measured by Townsend deprivation score, TDS, quartile)

	**TDS quartile 1 (least deprived)**	**TDS quartile 2**	**TDS quartile 3**	**TDS quartile 4 (most deprived)**
**Birthweight (mean, SD)**				
1961–1970	3297 (592)	3253 (617)	3213 (622)	3188 (613)
1971–1980	3345 (518)	3290 (536)	3240 (542)	3184 (565)
1981–1990	3373 (524)	3327 (549)	3264 (548)	3214 (568)
1991–2000	3405 (562)	3338 (581)	3270 (593)	3231 (587)
**Low birthweight (%)**				
1961–1970	7.2	9.0	10.6	10.7
1971–1980	5.1	6.2	7.5	9.2
1981–1990	4.6	5.6	6.8	8.6
1991–2000	5.1	6.6	8.3	9.0
**Preterm birth (%)**				
1961–1970	6.5	7.2	7.5	7.7
1971–1980	4.7	5.6	6.6	8.0
1981–1990	4.7	5.7	7.4	8.8
1991–2000	5.9	6.7	8.4	9.7

**Figure 2:**
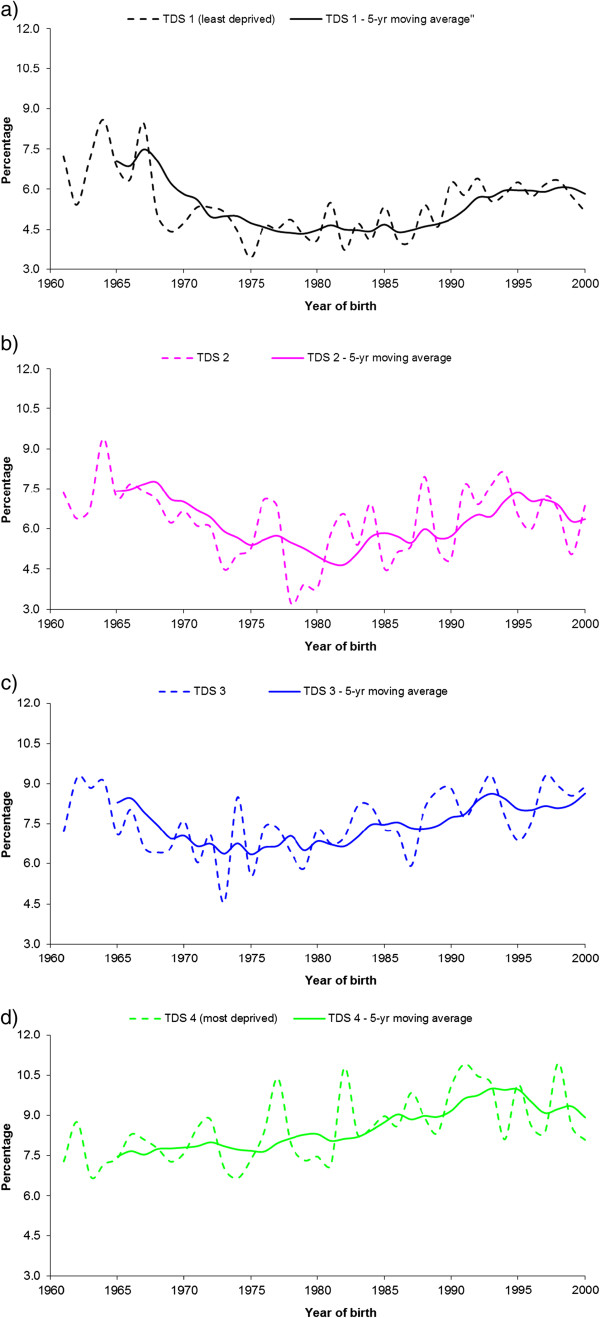
**The percentage of preterm birth by area deprivation measured by Townsend deprivation score (TDS) quartiles, Newcastle upon Tyne, 1961-2000: a) TDS 1 (least deprived); b) TDS 2; c) TDS 3; d) TDS 4 (most deprived).** The dotted lines are the observed (yearly) proportions for each TDS quartile, the solid lines are the five-year moving averages of the yearly proportions.

The percentage of LBW decreased from 10.7% in 1961 to 7.3% in 2000, a decrease of about 30%. The decrease in LBW was similar across all TDS quartiles (annual data not shown). The percentage of LBW and preterm birth for each TDS quartile by decade are shown in Table 1. The changes in preterm birth in relation to TDS quartiles are shown in Figure 2. For all TDS groups combined, there was little change in the percentage of preterm birth over four decades (7.5% in 1961 to 7.4% in 2000). In contrast, by TDS quartiles, there was a decrease in the percentage of preterm birth for Q1 (from 7.2% to 5.2%) and Q2 (from 7.4% to 6.9%) and an increase in the percentage of preterm birth for Q3 (from 7.2% to 8.9%) and Q4 (from 7.3% to 8.1%) over the study period (Figure 2). While the proportion of preterm births reduced by a quarter for the least deprived group (Q1), it increased by about the same proportion in Q3 group. This resulted in a marked socioeconomic gradient in the percentage of preterm birth by 2000, which was not observed in the early 1960-s.

There was a significant interaction between TDS quartile and decade of birth for birthweight (p = 0.028) but not for LBW (p = 0.277). Table 2 shows that SES gradients existed within each decade for both birthweight outcomes, but for LBW they were relatively smaller, which was reflected in the non-significant interaction result given above. For continuous birthweight, the SES gradient was smaller in the last decade when, compared to the first decade, the increase in birthweight was the most substantial for the most deprived group (Table 3). For preterm birth, there was no socioeconomic gradient in decade one in contrast to a clear gradient in later decades (Table 2), resulting in a highly significant interaction between TDS quartile and decade of birth (p < 0.001).

**Table 2:** Linear regression coefficients (95% confidence interval (95% CI)) for the change in birthweight and odds ratios (95% CI) for the change in the percentage of low birthweight and preterm birth by decade and by area deprivation group (Townsend deprivation score, TDS, quartile)

	**TDS quartile 1 (least deprived)**	**TDS quartile 2**	**TDS quartile 3**	**TDS quartile 4 (most deprived)**
**Birthweight**^**a**^				
1961–1970	Ref	–43.5 (–61.7, –25.2)	–69.5 (–88.5, –50.5)	–113.4 (–133.0, –93.8)
1971–1980	Ref	–35.6 (–50.6, –20.6)	–71.7 (–87.2, –56.3)	–109.0 (–125.0, –93.0)
1981–1990	Ref	–29.0 (–42.5, –15.5)	–71.5 (–85.7, –57.3)	–103.8 (–118.7, –89.0)
1991–2000	Ref	–42.9 (–57.0, –28.7)	–89.3 (–104.3, –74.4)	–97.5 (–113.0, –82.0)
**Low birthweight**^**a**^				
1961–1970	Ref	1.4 (1.2, 1.7)	1.6 (1.3, 1.9)	1.7 (1.5, 2.1)
1971–1980	Ref	1.1 (0.9, 1.3)	1.4 (1.1, 1.6)	1.5 (1.3, 1.8)
1981–1990	Ref	1.2 (1.0, 1.5)	1.4 (1.2, 1.7)	1.8 (1.5, 2.1)
1991–2000	Ref	1.3 (1.1, 1.6)	1.6 (1.4, 1.9)	1.6 (1.3, 1.9)
**Preterm birth**^**b**^				
1961–1970	Ref	1.1 (0.9, 1.2)	1.1 (0.9, 1.3)	1.1 (0.9, 1.3)
1971–1980	Ref	1.1 (0.9, 1.3)	1.3 (1.1, 1.5)	1.5 (1.3, 1.7)
1981–1990	Ref	1.2 (1.0, 1.4)	1.6 (1.4, 1.8)	1.8 (1.6, 2.1)
1991–2000	Ref	1.1 (0.9, 1.3)	1.3 (1.2, 1.5)	1.5 (1.3, 1.7)

^a^Adjusted for gestational age, maternal age, parity and infant sex.

^b^Adjusted preterm birth models did not include gestational age.

**Table 3:** Change in birthweight adjusted for gestational age by decade and by area deprivation group (Townsend deprivation score, TDS, quartile) with corresponding 95% confidence intervals (95% CI)

	**TDS quartile 1 (least deprived)**	**TDS quartile 2**	**TDS quartile 3**	**TDS quartile 4 (most deprived)**
**Change in birthweight (g) (95% CI)**^**a**^				
1961–1970	Ref	Ref	Ref	Ref
1971–1980	37.9 (22.4, 53.4)	43.4 (27.1, 59.6)	33.3 (16.5, 50.2)	32.3 (15.0, 49.5)
1981–1990	83.2 (67.8, 98.6)	105.7 (89.6, 121.8)	99.2 (82.6, 115.8)	107.3 (90.2, 124.3)
1991–2000	102.4 (86.7, 118.1)	107.4 (91.1, 123.7)	95.1 (78.6, 111.6)	124.9 (108.0, 141.9)

^a^Adjusted for gestational age, maternal age, parity and infant sex.

Table 3 shows that for each TDS quartile birthweight adjusted for gestational age increased throughout the four decades. The results were similar when the analysis was restricted to term births (data not shown).

## Discussion

This long-term population-based study from a relatively small conurbation with a stable population reports a clear socioeconomic gradient for continuous birthweight, LBW and preterm birth, with the higher risk of reduced birthweight, LBW and preterm birth in more deprived groups compared to the least deprived group using an area level deprivation measure. Over the four decades, mean birthweight increased and the percentage of LBW decreased across all deprivation groups. However, the percentage of preterm birth decreased in the least deprived groups and increased for the most deprived groups, thereby widening the gap between the most affluent and most deprived groups. There was a significant interaction between area deprivation and decade of birth for birthweight and preterm birth, while for LBW the SES gradients remained relatively smaller. In each SES group mean birthweight adjusted for gestational age (in all and term births) increased over time with a simultaneous decrease in the proportion of LBW, suggesting a possible increase in fetal growth across all TDS groups.

The findings of this study are consistent with those in similar studies across larger geographic areas in the United States, Canada, Europe, which report socioeconomic disparity for birthweight outcomes [15,18,25] and preterm birth [18,20,26,27,33,34]. Socioeconomic inequality in perinatal outcomes has been reported in both developing and developed countries [18,19,34-37], where socioeconomic gradients are lower and universal access to high quality prenatal and other medical care exists. Socioeconomic differences in birthweight and preterm birth have been observed in other British locations [7,13,20,36,38] as well as in other European [34,37,39] and non-European societies with different ethnic structure [18,20,26,40]. The inequalities in perinatal outcomes, particularly in fetal growth restriction, between more and less affluent socioeconomic groups are consistent across different societies irrespective of the measures of SES employed: individual, e.g. parental education [15], social class based on paternal occupation [41], or area-based measures [40,41]. Racial differences [15,40] or maternal demographic characteristics [15] cannot entirely explain the SES inequalities, although their contribution is substantial in locations with multiethnic populations. Despite an established association of SES with birthweight and/or preterm birth, only few population-based studies from European countries investigated temporal trends in the association of SES with these outcomes [34,36,37,39]. These studies, exploring trends during the last 20 years of the 20^th^ century, showed that the SES gap was either relatively stable or, similar to other health outcomes [23], was widening with time, despite temporal changes in society structure, maternal demographic characteristics and efforts to equalise access to medical care for all social groups. This tendency did not improve for very preterm birth (22–32 weeks) rate during 1994–2003 in the Trent health region, UK [13]. Our Newcastle study over the four decades, which were characterised by economy transformation in parallel with temporal changes in social structure of the population, demographic characteristics (maternal age and parity) and improvement in people’s living conditions, nutrition and prenatal care, found that the gap between most and least deprived groups did not narrow for birthweight and widened for preterm birth.

Socioeconomic inequality in birthweight is shown to be mediated by factors which can directly affect fetal growth, such as fetal exposure to maternal smoking [22]. The interaction between measures of socioeconomic and physical environment (i.e. traffic-related exposure) has been also demonstrated for birthweight, but less so for preterm birth [21]. The factors mediating the impact of socioeconomic inequality on birthweight may differ between developing and developed countries. Differences in nutrition and access to medical care may be more important in developing countries [2], whereas differences in maternal anthropometric characteristics, such as pre-pregnancy weight, BMI [42], lifestyle [22], and environmental exposure, such as air pollution [21], may be leading contributors in industrialised countries. The adjustment for such individual-level factors as gestational age, maternal age, parity and infant sex attenuated our results of reduced birthweight in all TDS quartiles compared to the reference least deprived quartile. The analysis of the association between mean maternal age by TDS quartile for each decade showed that in all four decades, mean maternal age was significantly higher for the least deprived group compared to the more deprived groups, gradually decreasing from the least deprived to the most deprived group in the last three decades (Additional file 1: Table S1). These differences in mean maternal age may have contributed to the socioeconomic inequality in birthweight, as younger maternal age is known to be associated with lower birthweight. However, the socioeconomic gradients in birthweight were evident after adjustment for maternal age in the model, suggesting the independent effect of SES on birthweight.

The above mediators of the association of SES with fetal growth are less important for preterm birth. A systematic review showed that maternal anthropometric features such as BMI, pre-pregnancy weight and maternal height were poor predictors of preterm delivery [43]. Cigarette smoking, a powerful mediator of the association between SES and fetal growth, may also mediate the relationship between SES and preterm birth, but to a lesser extent [2,44]. Other interrelated factors that may play a role in preterm birth, such as infection, e.g. bacterial vaginosis, and psychosocial stress [17,45], were suggested to be also linked with socioeconomic deprivation but the evidence for this is inconsistent [17].

The recent trend in increasing proportion of preterm birth observed in many countries may have resulted in part from more frequent obstetric interventions in compromised pregnancies at an earlier gestation as a result of improved survival of preterm babies. In this study, there was an increase in caesarean section from 1961 to 2000 [31], but the increase was similar across all four TDS groups (data not shown). Hence, the increase in the prevalence of preterm birth in the most deprived groups cannot be attributed to the higher increase in caesarean section compared to the least deprived groups in this population. A higher proportion of singleton pregnancies due to assisted conceptions may have contributed to the overall increase in the prevalence of preterm birth in the last decade of the study period [46], but it is more likely that their proportion was higher among women from advantaged SES groups and therefore cannot explain the rise in the prevalence of preterm birth in the more deprived groups.

This study has a number of strengths which have been described in detail elsewhere [31]. The validity of the long-term trends in birthweight and preterm birth with respect to area deprivation depends on the completeness and accuracy of the data across the study period, including gestational age estimation, which is one of the major strengths of the dataset [31]. Briefly, the accuracy of the data was ensured by multiple checking and internal and external cross-validation of the data. Estimation of gestational age was made as consistent as possible being mainly based on calculated values using the recorded estimated date of delivery (i.e. LMP based) throughout the study period rather than on the gestational weeks recorded in the neonatal records. The recorded gestational age may have been based on the ultrasound data for the later years if there was uncertainty in the LMP date or a significant discrepancy between the two estimates. Thereby we limited potential bias due to differences in sources for gestational age estimation over 40 years. Furthermore, we also demonstrated that temporal trends in birth numbers were similar to national and regional ones [31], which adds to the data validity. Data on gestational age and birthweight were missing for the majority of home births in the first decade of the study. Due to the lack of information from birth ledgers, it was not possible to assess whether there was a clear pattern in the distribution of home births by area deprivation in the early 1960s. As these births were not included in this analysis, data on birthweight and gestational age outcomes used in this study were compared across hospital births by decade and by TDS quartile (the percentage of home births in the last three decades of the study was very low, 1–2%). Perinatal mortality rates were lower among intended home births compared to hospital births in Newcastle upon Tyne during 1960–69 [47], suggesting that there was a lower percentage of LBW and preterm babies among them, but we were not able to prove this using our data.

In this study we used an area-based composite scoring method to identify the neighbourhood SES of the postcode area and assigned the same status to all mother/infant pairs residing within that postcode. Previous UK studies of birthweight outcomes showed that area-based TDS, as a proxy measure of SES, was a reliable alternative to individual measures [41]. Area deprivation measures are considered a better estimator of social gradient associated with birthweight [38,41]. A study from Newcastle found that TDS at the ED level was a good proxy for individual level deprivation to predict self-reported health [48]. Nevertheless, we cannot completely rule out misclassification of SES, whereby relatively deprived families may reside within affluent neighbourhoods or vice versa, but this effect is likely to be random. TDS calculation for 1997–2000 was based on data at ward level, a larger area than ED, which could have resulted in misclassification of births into TDS quartiles. However, the misclassification could occur in both directions and is unlikely to have a major impact on LBW and preterm birth rates in the last decade. The temporal trend of TDS for specific locations, and therefore the inequalities in TDS, may have been influenced by the implementation of local and national policies. An example for such trend is the implementation of the Housing Act 1980, which gave five million council house tenants in England and Wales the ‘Right to Buy’ their house from their local authority at a discount price. This policy may have favourably affected the TDS for a number of households in Newcastle upon Tyne for the two last decades, 1981–90 and 1991–2000, as home ownership is one of the variables for TDS calculation. However, a previous paper from Newcastle upon Tyne [31] reported that the gap between the most affluent and the most deprived groups of the population widened over the study period, which does not indicate that this policy substantially improved inequalities. We are not able to quantify the effect of the ‘Right to Buy’ policy on the TDS and the observed inequalities, but we can speculate that they would have been more pronounced if it was not implemented.

One of the study limitations was lack of data on maternal smoking, an important determinant of fetal growth, which did not allow us to examine the direct contribution of smoking to the association of SES with LBW and preterm birth. Information on some maternal anthropometric characteristics such as height, BMI or pre-pregnancy weight, which may have mediated the association between area-based deprivation and birthweight, but not preterm birth [43], was also not available in the routine neonatal records, a major data source of the study. We did not consider lack of another possible modifier of SES on birthweight or preterm birth, such as ethnic disparity [15,40], to be an important limitation of our study as Newcastle had a low percentage of ethnic minority groups (2%) [28].

## Conclusion

The study confirmed the role of socioeconomic inequalities on birthweight, LBW and preterm birth. The gap between the most and least deprived groups did not narrow for birthweight outcomes and widened for preterm birth over the four decades. These findings deserve due attention from a public health perspective. Further monitoring of changes in the association between SES and birth outcomes at the population level is important for prioritising public health interventions. Future studies are encouraged to use individual-level information on factors mediating the effects of socioeconomic inequality on birth outcomes to quantify their direct contribution and to determine the main target groups for interventions.

## Abbreviations

BMI: Body mass index; CI: Confidence interval; ED: Enumeration district; LBW: Low birthweight; LMP: Last menstrual period; OR: Odds ratio; PAMPER: Particulate Matter and Perinatal Events Research; SD: Standard deviation; SES: Socioeconomic status; TDS: Townsend deprivation score.

## Competing interests

The authors declare that they have no competing interests.

## Authors’ contributions

SVG, JR, MSP, LP and TPM contributed to the conception and design of the study, RG carried out the statistical analysis and SVG drafted the manuscript. All authors participated in the interpretation of the results, reviewed the paper for important intellectual content and read and approved the final version of the manuscript.

## Pre-publication history

The pre-publication history for this paper can be accessed here:

http://www.biomedcentral.com/1471-2458/13/345/prepub

## Supplementary Material

Additional file 1: Table S1 Mean (SD) maternal age (years) by decade of birth and by area deprivation group (Townsend deprivation score, TDS, quartile). Click here for file
