# Supplementary material for: No improvement in socioeconomic inequalities in birthweight and preterm birth over four decades: a population-based cohort study
Source: BMC Public Health. 2013 Apr 15;13:345. doi: 10.1186/1471-2458-13-345 (PMC3651338; doi:10.1186/1471-2458-13-345)
Supplement: Additional file 1: Table S1 — Mean (SD) maternal age (years) by decade of birth and by area deprivation group (Townsend deprivation score, TDS, quartile). [file 1471-2458-13-345-S1.doc]

**Additional file 1: Table S**1. Mean (SD) maternal age (years) by decade of birth and by area deprivation group (Townsend deprivation score, TDS, quartile).

| **TDS Quartile** | **1961-1970*** | **1971–1980*** | **1981–1990*** | **1991-2000*** |
| --- | --- | --- | --- | --- |
| Quartile 1 (least deprived) | 27.5 (5.9) | 27.1 (4.6) | 28.3 (4.5) | 30.3 (4.8) |
| Quartile 2 | 26.4 (6.2) | 25.1 (5.0) | 26.3 (5.1) | 27.9 (5.5) |
| Quartile 3 | 26.1 (6.3) | 24.1 (5.2) | 24.3 (5.0) | 25.5 (5.6) |
| Quartile 4 (most deprived) | 26.4 (6.8) | 23.4 (5.3) | 23.5 (4.8) | 24.7 (5.5) |

*p<0.001 for the difference in mean maternal age between the TDS quartiles in each decade, using one-way analysis of variance.
